# How Children’s Cognitive Reflection Shapes Their Science Understanding

**DOI:** 10.3389/fpsyg.2020.01247

**Published:** 2020-06-24

**Authors:** Andrew G. Young, Andrew Shtulman

**Affiliations:** ^1^Department of Psychology, Northeastern Illinois University, Chicago, IL, United States; ^2^Department of Psychology, Occidental College, Los Angeles, CA, United States

**Keywords:** conceptual development, scientific reasoning, explanatory coexistence, intuitive theories, cognitive reflection

## Abstract

Learning science requires contending with intuitions that are incompatible with scientific principles, such as the intuition that animals are alive but plants are not or the intuition that solids are composed of matter but gases are not. Here, we explore the tension between science and intuition in elementary school–aged children and whether that tension is moderated by children’s tendency to reflect on their intuitions. Our participants were children between the ages of 5 and 12 years (*n* = 142). They were administered a statement-verification task, in which they judged statements about life and matter as true or false, as well as a children’s Cognitive Reflection Test (CRT-D), in which they answered “brain teasers” designed to elicit an intuitive, yet inaccurate, response that could be corrected upon further reflection. Participants also received a tutorial on the scientific properties of life or matter, sandwiched between two blocks of the statement-verification task. We found that performance on the statement-verification task, which pitted scientific conceptions against intuitive conceptions (e.g., “cactuses are alive”), was predicted by performance on the CRT-D, independent of age. Children with higher levels of cognitive reflection verified scientific statements more accurately before the tutorial, and they made greater gains in accuracy following the tutorial. These results indicate that children experience conflict between scientific and intuitive conceptions of a domain in the earliest stages of acquiring scientific knowledge but can learn to resolve that conflict in favor of scientific conceptions, particularly if they are predisposed toward cognitive reflection.

## Introduction

Our first theories of natural phenomena – intuitive theories – are often incompatible with the scientific theories we learn later in life. They are developed by children from a combination of inputs, including innate biases, firsthand experience, and cultural teachings (Vosniadou, 1994; Carey, 2009; Shtulman, 2017), and they play the same inferential role as scientific theories, helping us explain past events, predict future events, and intervene on present events (Gopnik and Wellman, 2012). But they differ from scientific theories in that they carve up the world into entities and processes that do not align with the true causes of natural phenomena.

One well-studied example of intuitive theories is children’s theories of life (Stavy and Wax, 1989; Hatano and Inagaki, 1994; Slaughter and Lyons, 2003). Life is a metabolic state – the consumption of energy to further an organism’s survival and reproduction – but young children do not know of the internal structures that make metabolism possible, so they interpret life as related to motion instead. Preschoolers classify moving but non-living entities, such as the sun and the clouds, as alive and classify living but non-moving objects, such as plants and trees, as not alive. These mistakes persist until children conceive of life as supported by the interrelated functions of internal organs, typically by age 10 years. Young children understand that organisms must eat and sleep in order to move and grow, but they lack the physioanatomical knowledge needed to conceive of them as bodily machines.

Another well-studied example is children’s theories of matter (Carey, 1991; Nakhleh et al., 2005; Smith, 2007). Matter is anything composed of atoms, but most material substances betray no perceptible sign of their composition. Gases and vapors are all composed of atoms, but children can neither see them nor hold them, so they classify them as non-material. They also deny that such substances have weight or take up space. Children also make the converse mistake of classifying non-material entities that they can see or feel as matter, including echoes, shadows, and heat. This pattern persists until early adolescence, when children learn a particulate theory of matter in introductory physical science.

In the present study, we assess how children reason about life and matter in relation to their *cognitive reflection*, or their tendency to reflect on their own thinking. Learning about life and matter requires recognizing that one’s intuitive understanding of a domain is incompatible with a scientific understanding, as well as the ability to suppress the former in favor of the latter. Children who are disposed to reflect on their own thinking may have an advantage at these tasks relative to those who are not. By studying how children’s cognitive reflection relates to their understanding of counterintuitive scientific ideas, we shed new light on the domain-general resources that allow children to construct domain-specific theories of the natural world.

### Explanatory Coexistence

Learning a scientific theory at odds with an intuitive theory requires conceptual change, or knowledge revision at the level of individual concepts. Conceptual change has traditionally been viewed as a process of restructuring and replacement (Carey, 1985; Nersessian, 1989; Chi, 1992; Vosniadou, 1994). Intuitive theories are restructured to accommodate counterintuitive scientific information and thus erased in the process, in the same way that remodeling a house erases the footprint of its original layout.

This view has been challenged by research revealing that intuitive theories continue to influence scientific reasoning throughout the life span, particularly when reasoners are cognitively burdened or cognitively impaired. In the domain of life, for instance, college undergraduates instructed to classify entities as “alive” or “not alive” under time pressure are prone to make the same mistakes that preschoolers make, classifying moving but non-living things as alive and living but non-moving things as not alive (Goldberg and Thompson-Schill, 2009). Alzheimer’s disease patients make the same mistakes, even when given ample time to respond, and they explicitly define life in terms of motion rather than metabolic activity (Zaitchik and Solomon, 2008). Even elderly adults without Alzheimer’s disease are inclined to conflate life with self-directed motion (Tardiff et al., 2017), indicating that childhood misconceptions persist across the life span and must be inhibited when reasoning about life as a metabolic process.

Early intuitions about matter also reemerge under cognitive load. Adults instructed to decide whether something is material or non-material as quickly as possible will mistakenly classify gases and heft-less objects, such as dust and snowflakes, as non-material and mistakenly classify perceptible forms of energy, such as rainbows and lightning, as material (Shtulman and Legare, 2020). Adults also make systematic mistakes in deciding whether an object will sink or float. When shown two balls of equal size, one made of wood and one made of lead, they judge that the wood ball is more likely to float than the lead one. But shown a large ball of wood and a small ball of lead, they take reliably longer to make the same judgment (Potvin et al., 2015; Potvin and Cyr, 2017).

Research over the past decade has revealed that this pattern is widespread (Shtulman and Lombrozo, 2016). Adults verify counterintuitive scientific ideas more slowly and less accurately than closely matched intuitive ones in several domains, including astronomy, genetics, mechanics, thermodynamics, and evolution (Shtulman and Valcarcel, 2012; Shtulman and Harrington, 2016). And these effects have been observed in several populations, including high schoolers (Babai et al., 2010), undergraduate science majors (Foisy et al., 2015), high school science teachers (Potvin and Cyr, 2017), and elderly adults (Barlev et al., 2018). Even professional physicists (Kelemen et al., 2013) and professional biologists (Goldberg and Thompson-Schill, 2009) exhibit cognitive conflict when reasoning about counterintuitive scientific ideas. Such conflict indicates that early intuitions about natural phenomena survive the acquisition of contradictory scientific knowledge.

### Cognitive Reflection

The current study investigates whether learning counterintuitive scientific ideas is shaped by cognitive reflection or the disposition to reflect on, and override, our first intuition. This disposition is most commonly measured by the Cognitive Reflection Test (CRT; Frederick, 2005), a three-item test designed for adults. Consider this item: “In a lake, there is a patch of lily pads. Every day, the patch doubles in size. If it takes 48 days for the patch to cover the entire lake, how long would it take for the patch to cover half of the lake?” The correct answer is 47, given the patch must have covered half the lake a day prior to covering the entire lake, but the question is designed to elicit an intuitive response of 24, or half of 48.

Adults who perform well on the CRT demonstrate superior performance on many other reasoning tasks, including those measuring logical reasoning, probabilistic reasoning, argumentation, and temporal discounting (Frederick, 2005; Toplak et al., 2011). The CRT is a stronger predictor of performance on such tasks than either general intelligence or executive functioning (Toplak et al., 2011). The CRT also correlates with causal reasoning (Don et al., 2016), moral reasoning (Royzman et al., 2014), endorsement of scientific claims (Gervais, 2015), rejection of supernatural claims (Pennycook et al., 2012), rejection of stereotypes (Hammond and Cimpian, 2017), and detection of fake news (Pennycook and Rand, 2018).

Cognitive reflection has been studied extensively in adults, but little is known about its development. To address this gap, we created a nine-item CRT for school-aged children, the CRT–Developmental Version (CRT-D). Each item elicits an intuitive, but incorrect, lure response that school-aged children should be able to correct upon further reflection. In a preliminary study (Young A. G. et al., 2018), we found that adults’ performance on the CRT-D was strongly correlated with their performance on the original CRT, as well as their performance on various heuristics-and-biases tasks, and children’s performance on the CRT-D was strongly correlated with child-friendly versions of the same tasks, even when controlling for age.

### Research Objectives

Prior research indicates that cognitive reflection supports science understanding in adults (Shtulman and McCallum, 2014). College students’ CRT scores predict their understanding of astronomy, evolution, geology, mechanics, perception, and thermodynamics more strongly than their prior STEM coursework, their statistical reasoning ability, or their understanding of the nature of science. Here, we explore whether CRT scores predict science understanding in children, who are in the earliest stages of learning science and who have less experience reflecting on their own cognition.

We also explore whether cognitive reflection predicts children’s ability to learn new scientific information, by providing them with tutorials on life and matter. In previous research (Young A. et al., 2018), we found that such tutorials promote adults’ ability to verify counterintuitive scientific ideas. Adults are reliably slower and less accurate at verifying counterintuitive statements such as “dust has weight” and “yeast needs nutrients” relative to intuitive statements involving the same predicates, such as “bricks have weight” and “goats need nutrients.” Providing adults with tutorials on the scientific properties of life and matter helped them close the gap in accuracy between the two types of statements but not the gap in latency. In other words, the tutorials did not assuage the immediate conflict elicited by counterintuitive statements (as indexed by response times), but they did help participants favor scientific responses over intuitive ones.

In the present study, we extended this line of research to elementary schoolers. Our study followed the same protocol as Young A. et al. (2018), which included a pretest, a tutorial, and a posttest. It expanded on that protocol by including a domain-general measure of cognitive ability, the CRT-D. We expected children to show signs of conflict between science and intuition, given that the children in our age range had begun to learn about life and matter in school, and we expected children to verify counterintuitive statements more accurately following instruction. It was an open question, though, whether children’s performance on the statement-verification task would correlate with their performance on the CRT-D or whether improvements in performance, from pretest to posttest, would correlate as well.

## Methods

### Participants

Our participants were 142 children in kindergarten through 6th grade. Their mean age was 8 years and 5 months, and they were approximately balanced for gender (82 female, 62 male). Children were recruited from public playgrounds and a children’s museum, and they completed the study onsite.

### Materials

#### Cognitive Reflection Test – Developmental Version

Children answered the nine cognitive reflection questions in Table 1 (from Young A. G. et al., 2018). We used the number of correct responses as children’s score, with higher scores indicating greater cognitive reflection (mean = 2.8, *SD* = 1.9, range = 0–8). Reliability for the measure was acceptable (McDonald’s ω total = 0.74). While some children may have lacked the knowledge or cultural background required to answer certain items correctly, we took a conservative approach and retained all CRT-D items because they matched the response structure of the original CRT, namely, they elicited more intuitive responses than other incorrect responses. Ongoing research aims to verify that CRT-D items are functioning as intended across diverse samples.

**TABLE 1:** Items on the Cognitive Reflection Test–Developmental Version (CRT-D), along with their correct answer and the intuitive answer they were designed to prime.

**Item**	**Correct**	**Intuitive**
If you’re running a race and you pass the person in second place, what place are you in?	Second	First
Emily’s father has three daughters. The first two are named Monday and Tuesday. What is the third daughter’s name?	Emily	Wednesday
A farmer has 5 sheep, all but 3 run away. How many are left?	Three	Two
If there are 3 apples and you take away 2, how many do you have?	Two	One
What do cows drink?	Water	Milk
What weighs more, a pound of rocks or a pound of feathers?	Same weight	Rocks
What hatches from a butterfly egg?	Caterpillar	Butterfly
Who makes Christmas presents at the North Pole?	Elves	Santa
Anna is playing foursquare with her three friends: Eeny, Meeny, and Miny. Who is the fourth player?	Anna	Mo

#### Statement-Verification Task

We measured children’s understanding of counterintuitive scientific ideas with a statement-verification task. Children were asked to judge four types of statements as true or false. Some statements were true from both a scientific perspective and an intuitive perspective (“tigers need nutrients”); some were false from both perspectives (“forks need nutrients”); some were true from a scientific perspective but false from an intuitive perspective (“bacteria need nutrients”), and some were false from a scientific perspective but true from an intuitive perspective (“fire needs nutrients”). The first two types of statements will be referred to as intuitive, and the latter two types as counterintuitive.

For each domain, statements were generated by pairing three predicates with 32 entities. In the domain of life, the predicates were “reproduces,” “needs nutrients,” and “grows and develops.” These predicates apply to all living things, but we predicted children would be more inclined to apply them to entities that exhibit self-directed motion. In the domain of matter, the predicates were “has weight,” “takes up space,” and “is made of atoms.” These predicates apply to all material things, but we predicted children would be more inclined to apply them to entities that can be seen or felt. Because children might not know the meanings of certain predicates, we defined each on first introduction. “Reproduce,” for instance, was defined as “things that can make more things like themselves,” and “made of atoms” was defined as “things that are made of up of tiny pieces.”

Predicates were paired with the four types of entities shown in Table 2. In the domain of life, those entities were animals (deemed alive by both science and intuition), inanimate artifacts and inanimate natural kinds (deemed alive by neither science nor intuition), plants and microorganisms (deemed alive by science but not intuition), and animate natural kinds (deemed alive by intuition but not science). In the domain of matter, those entities were physical objects (deemed material by both science and intuition), abstract ideas (deemed material by neither science nor intuition), gases and other substances lacking bulk or heft (deemed material by science but not intuition), and the visible or tangible components of energy (deemed material by intuition but not science). These pairings created the four types of statements described above: statements deemed true by both science and intuition (“bricks take up space”), statements deemed false by both science and intuition (“dreams take up space”), statements deemed true by science but not intuition (“air takes up space”), and statements deemed true by intuition but not science (“rainbows take up space”).

**TABLE 2:** Sample items used in the biological statements (top) and physical statements (bottom) on the statement-verification task, organized by their classification according to science and intuition.

	**Intuitive classification**	
**Scientific classification**	**Living**	**Non-living**
Living	Rabbits	Mushrooms
	Turtles	Grass
	Snails	Bacteria
Non-living	Sun	Hammers
	Wind	Caves
	Fire	Shells

	**Intuitive classification**	
**Scientific classification**	**Material**	**Non-material**

Material	Bricks	Smoke
	Ice	Snowflakes
	Logs	Air
Non-material	Rainbows	Dreams
	Shadows	Songs
	Heat	Numbers

Children completed the task on an iPad, responding via touch screen. Fifty children opted into a version of the task that played audio recordings of the statements, obviating the need to read the statements. Audio recordings of each statement were generated using Apple’s macOS text-to-speech engine. Children who listened to the audio-recorded stimuli received only four of the six predicates, due to the additional time required to play the recordings.

#### Tutorials

Children completed a tutorial on life or matter midway through the experiment. Each tutorial began with definitions of key characteristics of the domain, followed by a brief video that illustrated those characteristics with examples. The tutorials then addressed common misconceptions about the domain, followed by videos that illustrated why these misconceptions were false. The tutorial on life emphasized that all living things need energy and nutrients, grow and develop, react to stimuli in their environment, and reproduce. It addressed the misconception that life is synonymous with self-directed motion with examples of entities that do not move but are alive (e.g., moss) and entities that move but are not alive (e.g., comets). The tutorial on matter emphasized that all matter occupies space, has weight, is made of atoms, and can undergo phase transitions. It addressed the misconception that matter is synonymous with perceptibility with examples of entities that cannot be perceived but are material (e.g., gases) and entities that can be perceived but are not material (e.g., lightning). Tutorials took approximately 7 min to complete.

### Procedure

Children completed the CRT-D, then verified 48 statements about life and 48 statements about matter (pretest), then completed a tutorial on life or matter, and finally verified 48 additional statements from each domain (posttest). Children were randomized to tutorial condition in equal proportions.

Children completed the pretest and posttest in blocks. They saw a screen introducing a particular predicate (“Does it grow and develop?”), followed by 16 statements with that predicate (“Seaweed grows and develops”). The statements were randomly ordered within a block, and the blocks were randomly ordered within the testing phase, meaning that biological and physical predicates were intermixed.

Children saw the same predicates at pretest and posttest, but those predicates were paired with 16 new items; that is, the 48 statements children saw at pretest (3 predicates × 16 items) were different from the 48 statements they saw at posttest. Items were randomly assigned to one of two item sets for counterbalancing, so that the 48-statement pretest for some children constituted the 48-statement posttest for other children and *vice versa*. Because of experimenter error, item sets were imperfectly balanced. One set was presented at pretest for 73% of children, whereas the other was presented at pretest for 27% of children. Preliminary analyses found item set was not a significant predictor of speed or accuracy, by itself or in interaction with statement type, in either domain at either test period, indicating that children’s performance was consistent across item sets.

## Results

The statement-verification task yielded two outcome measures: response accuracy and response latency. We analyzed each outcome with a linear mixed model (LMM), with statement type (intuitive or counterintuitive), test (pretest or posttest), instruction (instructed or uninstructed), and their interactions as fixed effects and by-participant and by-predicate random effects. Models with maximal random effects structures had convergence issues, and thus we followed the procedure recommended by Bates et al. (2015) to guide removal of random effects that were not supported by the data. Inference for fixed effects was carried out via type 3 likelihood ratio test (LRT) model comparison.

Our primary analyses collapse across tutorial domain (life or matter) and focus on whether the statements were targeted by instruction or not. Response latencies were similar across domains (2.83 vs. 2.93 s), and effects of instruction were similar across domains. But children did verify biological statements more accurately than physical statements (85 vs. 74%), so we report domain-specific results when analyzing response accuracy.

As noted above, some children listened to the scientific statements, and some read them on their own. The latter responded more quickly (2.30 vs. 3.96 s) and more accurately (82 vs. 74%) and were also older (9;1 vs. 7;2) and higher in cognitive reflection (3.2 vs. 2.1). However, a parallel set of models that adjusted for presentation format (read or listen) and its interactions with other predictors yielded similar findings to the reported results.

### Response Accuracy

**FIGURE 1:**
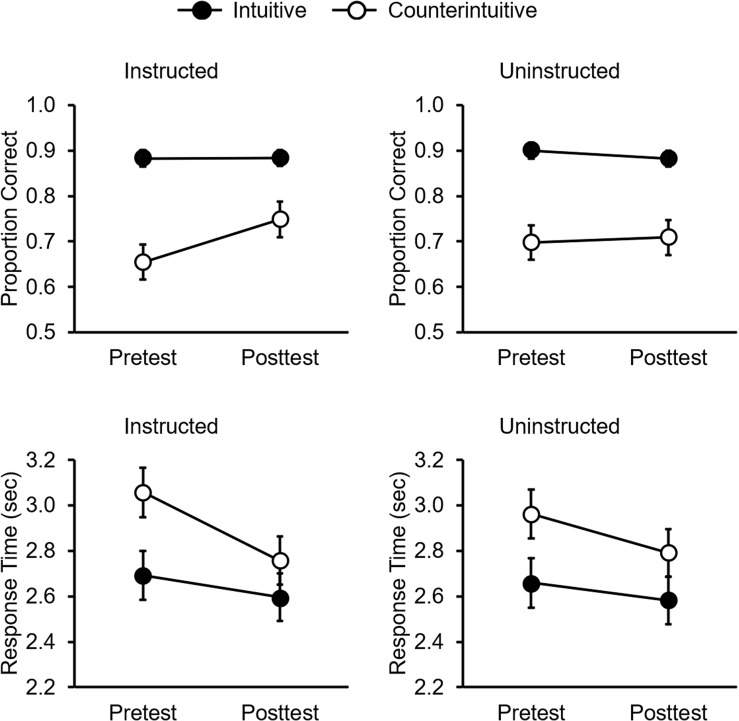
Estimated proportion of correct verifications **(top)** and response latency **(bottom)** by statement type, test, and instruction. Error bars represent standard errors.

As seen in Figure 1, there was an effect of statement type, such that children verified intuitive statements more accurately than counterintuitive statements, LRT χ^2^(1) = 10.46, *p* < 0.001. Overall, accuracy for intuitive statements was 18.5% greater than accuracy for counterintuitive statements, 95% confidence interval (CI) [12.1, 24.9]. Additionally, there was a three-way interaction between statement type, test period, and instruction, LRT χ^2^(1) = 11.15, *p* < 0.001. In the instructed domain, children’s posttest accuracy for counterintuitive statements was 9.4% greater than their pretest performance, 95% CI [7.1, 11.6]. This learning was observed in both the life domain, 95% CI [1.7, 8.1], and matter domain, 95% CI [10.6, 16.8]. This effect was limited to counterintuitive statements in the instructed domain; intuitive statements were verified with similar accuracy at pretest and posttest in both domains, as were counterintuitive statements in the uninstructed domain. Instruction was thus effective at improving children’s accuracy at verifying counterintuitive scientific ideas within the targeted domain.

### Response Latency

Following prior research, we analyzed response latencies for correctly verified statements only. Response latency thus indicates whether arriving at a correct response entailed more cognitive conflict for some statements relative to others. Before doing so, we first removed latencies shorter than 250 ms, as they were too quick to have been deliberate. We then calculated the mean response latency across participants and statements (mean = 2,968 ms) and removed latencies more than 2 SDs above the mean (>9,071 ms).

As seen in Figure 1, there was an effect of statement type, such that children correctly verified counterintuitive statements more slowly than intuitive ones, LRT χ^2^(1) = 102.61, *p* < 0.001. Response latencies for counterintuitive statements were 260 ms slower than response latencies for intuitive statements, 95% CI [210, 309]. Additionally, there was an interaction between test and statement type, LRT χ^2^(1) = 8.79, *p* = 0.003. Children correctly verified counterintuitive statements 198 ms faster at posttest than pretest, 95% CI [43, 352], but response latencies for intuitive statements were similar at pretest and posttest. We suspect this effect was due to increased familiarity with the task and greater initial latencies at pretest, as it did not vary by instruction [three-way interaction: LRT χ^2^(1) = 1.18, *p* = 0.278].

### Cognitive Reflection

Children’s CRT-D performance was moderately to strongly correlated with response accuracy at both test periods for both types of statements in both domains (*r*’s = 0.27–0.53, *p*’s < 0.002). These correlations indicate that children with higher CRT-D scores performed more accurately on the statement-verification task across the board, but did they also learn more from instruction?

**FIGURE 2:**
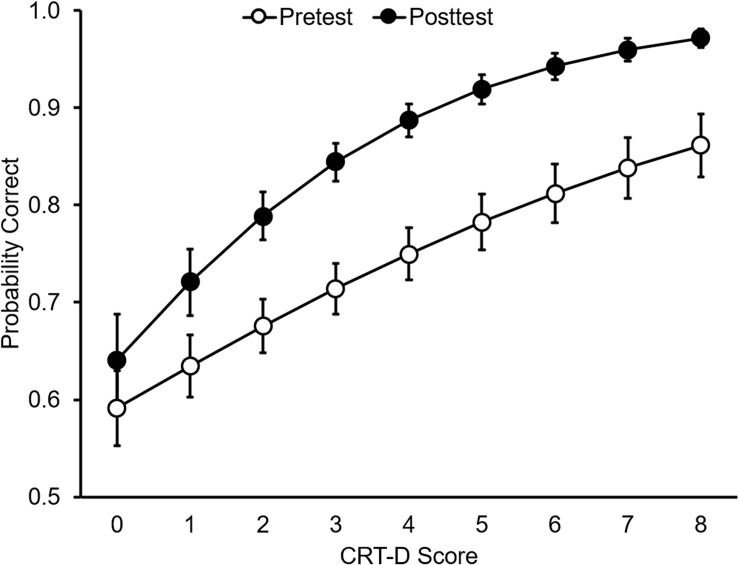
Estimated probability of correct verifications by CRT-D score and test. Error bars represent standard errors.

We estimated a binomial generalized LMM on children’s correct responses for counterintuitive statements in the instructed domain with test (pretest or posttest), CRT-D score, and their interaction as fixed effects and by-participant and by-item random effects. This analysis revealed an interaction between test and CRT-D score, LRT χ^2^(1) = 12.19, *p* < 0.001. Children with higher CRT-D scores showed larger gains in accuracy from pretest to posttest, logit *b*_*Test* × *CRT.D*_ = 0.19, 95% CI [0.08, 0.29], as shown in Figure 2. This result was observed in both the life domain, 95% CI [0.00, 0.32], and matter domain, 95% CI [0.08, 0.34]. Critically, the interaction between test and CRT-D score remained significant in an additional model that included fixed effects for age and an age-by-test interaction, LRT χ^2^(1) = 4.30, *p* = 0.038.

Children’s CRT-D performance yielded moderate to strong negative correlations with response latencies at both test periods for both types of statements in both domains (*r*’s = −0.39 to −0.48, *p*’s < 0.001). We explored the potential effects of CRT-D performance by estimating an LMM with statement type (intuitive or counterintuitive), test (pretest or posttest), instruction (instructed or uninstructed), CRT-D, and their interactions as fixed effects and by-participant and by-predicate random effects. There was an overall effect of CRT-D performance, LRT χ^2^(1) = 24.14, *p* < 0.001, such that children with greater cognitive reflection had shorter latencies, but no interactions involving CRT-D were observed.

## Discussion

Do elementary schoolers exhibit cognitive conflict when reasoning about counterintuitive scientific ideas? And does their tendency to reflect on their own cognition moderate this conflict? Our findings support both possibilities. Children were slower and less accurate at verifying scientific statements that conflict with their intuitive theories of life or matter compared to closely matched statements that accord with those theories. Instructing children on the scientific properties of life or matter increased their accuracy for counterintuitive statements in the instructed domain but did not increase their speed (relative to intuitive statements). These findings indicate that children experience conflict between scientific ideas and intuitive ideas, despite limited exposure to science, but this conflict can be resolved in favor of scientific ideas with targeted instruction.

Children’s accuracy at verifying domain-specific scientific statements was predicted by a domain-general disposition: cognitive reflection. Cognitively reflective children were more accurate at verifying scientific statements at both pretest and posttest. They also learned more from instruction, exhibiting larger gains in accuracy from pretest to posttest than children with lower CRT-D scores.

Our findings parallel those of Young A. et al. (2018), who administered the same task to adults. The adults were faster and more accurate than children in the present study, but both groups verified counterintuitive statements more slowly and less accurately than closely matched intuitive statements. The effect of instruction was also similar across age groups, increasing participants’ accuracy at verifying counterintuitive statements but not their speed. Cognitive conflict between science and intuition thus appears to take the same form across development.

Our findings also parallel those documented by Vosniadou et al. (2018), who assessed tensions between children’s intuitive and scientific reasoning in a different task. These researchers asked third- and fifth-graders to sort physical and biological items into one of two categories: a category that emphasized the item’s intuitive features or a category that emphasized its scientific features. For instance, participants decided whether water should be grouped with other liquids (coke, lemonade, milk) or with other forms of H_2_O (ice, vapor, snow). Children of all ages preferred intuitive categories over scientific categories, and they took longer to make their judgments when they opted for the scientific category instead.

Vosniadou et al. also measured children’s executive function skills – set-shifting ability and inhibitory control – and found that children with higher executive function were more likely to categorize the target items by their scientific properties and were faster to do so (see also Bascandziev et al., 2018; Tardiff et al., 2020). These findings echo our finding that children with higher cognitive reflection were more accurate at verifying counterintuitive scientific ideas, and they raise questions about the relation between cognitive reflection and executive function. Cognitive reflection draws on similar skills, as children must inhibit a gut response (inhibition) in order to shift to another response (set shifting) while holding the question in mind (working memory). But succeeding on the CRT-D also requires recognizing that such activities are necessary, as well as the ability to coordinate them on one’s own. The “stop and think” aspect of cognitive reflection may transcend the individual components of executive function. Studies of rational thought have found that cognitive reflection predicts performance on heuristics-and-biases tasks independent of executive function (Toplak et al., 2011; Young and Shtulman, 2020), but future research is needed to determine whether the same is true of science understanding and, if so, which aspects of science understanding are uniquely predicted by cognitive reflection.

The current findings suggest that cognitive reflection may be a prerequisite for changing certain cognitive representations, but it remains unclear as to why. Cognitively reflective individuals may be better at identifying gaps in their understanding, or they may be better at filling those gaps with new information. They may be more receptive to instruction, or they may be better at monitoring and resolving response conflicts. We suspect that cognitive reflection is valuable because it fosters metaconceptual awareness. All children reason *with* their concepts, but they might not reason *about* their concepts, and this latter ability may be required for changing them. Pedagogically, our findings imply that instructors could use the CRT-D as a diagnostic for determining who is likely to profit from instruction and who is not. Children with low CRT-D scores may benefit from more instruction, or different instruction, than their peers.

In conclusion, we have shown that conflict between science and intuition emerges early in the acquisition of scientific knowledge. Children in the earliest stages of science education verify scientific ideas that conflict with their intuitive theories more slowly and less accurately than those that accord with them. Although this conflict may be inevitable, children can learn to privilege scientific ideas over intuitive ones with instruction that challenges intuitive theories and a disposition toward questioning intuitive responses.

## Data Availability Statement

The datasets for this study are available at https://osf.io/rxw27/.

## Ethics Statement

The studies involving human participants were reviewed and approved by the Occidental College Institutional Review Board. Written informed consent to participate in this study was provided by the participants’ legal guardian/next of kin.

## Author Contributions

Both authors contributed to all aspects of the data collection, the data analysis, and the write up.

## Conflict of Interest

The authors declare that the research was conducted in the absence of any commercial or financial relationships that could be construed as a potential conflict of interest.
